# Self-assembled/composited lignin colloids utilizing for therapy, cosmetics and emulsification

**DOI:** 10.3389/fchem.2022.1107643

**Published:** 2022-12-21

**Authors:** Yating Bai, Xing Wang, Xinru Wang, Xujie Yang, Xinke Li, Hanwen Xin, Dayin Sun, Jinghui Zhou

**Affiliations:** ^1^ Liaoning Key Lab of Lignocellulose Chemistry and BioMaterials, Liaoning Collaborative Innovation Center for Lignocellulosic Biorefinery, College of Light Industry and Chemical Engineering, Dalian Polytechnic University, Dalian, China; ^2^ Polymer Institute of Science and Engineering, Department of Chemical Engineering, Tsinghua University, Beijing, China

**Keywords:** lignin colloids, self-assembly, composite, therapy, cosmetics, emulsification

## Abstract

Lignin, the most abundant source of renewable aromatic compounds on the planet, is attracting more scholarly attention due to its possibility of replacing petroleum-based chemicals and products. However, it remains underutilized because of the heterogeneity of its multi-level structure that prevents homogenization and standardization of derived products. The key to solving these problems lies in finding a general preparation method to achieve the integrated utilization of lignin molecules at all levels. The assembly-mediated granulation methods provide a significant means for the integrated value-added utilization of lignin, and for biomass productization applications, it is significant to understand the molecular mechanisms of lignin nano-colloids (LNCs) formation thus accurately guiding their functionalization. Therefore, a thorough understanding of the assembly morphology and behavior of lignin in different solutions towards colloids is of great scientific importance. In this minireview, we focus on the assembly behavior of lignin in different solvents, specifically in mono-solvent and multi-solvent, and in particular, we review various methods for preparing lignin composite colloids and concentrate on the applications in therapy, cosmetics and emulsification, which are important for guiding the preparation and efficient utilization of LNCs.

## Graphical Abstract

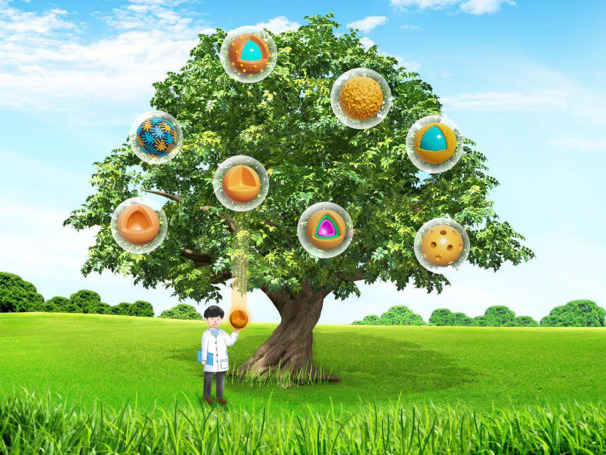

## 1 Introduction

The word “lignin” is derived from the Latin word “lignum”. It was first used by the Swiss botanist A. P. Candolle (1778–1841) to refer to “wood” (Calvo-Flores and Dobado, 2010). Lignin and cellulose/hemicellulose, as important components of biomass resources, have appeared in different forms and roles at various stages of human species evolution and have accompanied the development of human civilization (Liu et al., 2021; Liu R. et al., 2022). Lignocellulosic biomass is considered to be a renewable resource that can be used to produce biofuels or chemicals through different biorefining processes. Lignocellulosic biomass consists primarily of cellulose, hemicellulose and lignin as the main structural biopolymer. Lignin is of special interest because of its aromatic structure, which possesses the potential to replace industrially relevant aromatic polymers and fine chemicals (Xu et al., 2021; Xu J. et al., 2022). As the most abundant renewable phenolic biopolymer on earth, lignin is likely to replace petroleum-based aromatic polymers. Stabilization of lignin could reduce the carbon footprint and achieve a sustainable economy (Balakshin et al., 2020; Zhou et al., 2021). However, there are still huge challenges for the integrated use of lignin, and so far no abundant commercial lignin products have been created except for common products such as sodium lignosulfonate, an emerging field with huge production capacity (Zhou et al., 2021). It is well known that there is significant heterogeneity and heterogeneity in the molecular composition and structure of lignin macromolecules at all levels, making processing extremely difficult (Wijaya et al., 2021). Therefore, exploring and developing a general preparation route for residue-free synthesis would open up a new era in the utilization of lignin as a whole component. Obviously, granulation is a convenient route.

LNCs are nanoparticles constructed with lignin as the main component, which can be formed by self-assembly of lignin macromolecules in a solvent or prepared by other granulation methods, to achieve the compound of functional substances and functional expansion (Lizundia et al., 2021). Lignin possesses the advantage of being biodegradable and biocompatible, showing that it can be a green tool. LNCs carry drugs, genes and proteins through their strong non-bonded interactions for anti-microbial or disease treatment (Richter et al., 2015; Figueiredo et al., 2017; Sipponen et al., 2019). In addition to their use as nanocarriers for biomedical needs, LNCs possess great potential for skin health (Qian et al., 2014b; Qian et al., 2015b; Trevisan and Rezende, 2020), Pickering emulsion systems (Agustin et al., 2019; Bertolo et al., 2019; Li et al., 2021) and filler (Qian et al., 2015a) applications. However, no matter which field they are applied to, the aggregation and assembly form of LNCs play an important role. Without the aggregation and assembly, it is not possible to modify LNCs with specific functions or to form homogeneous LNCs.

**FIGURE 1:**
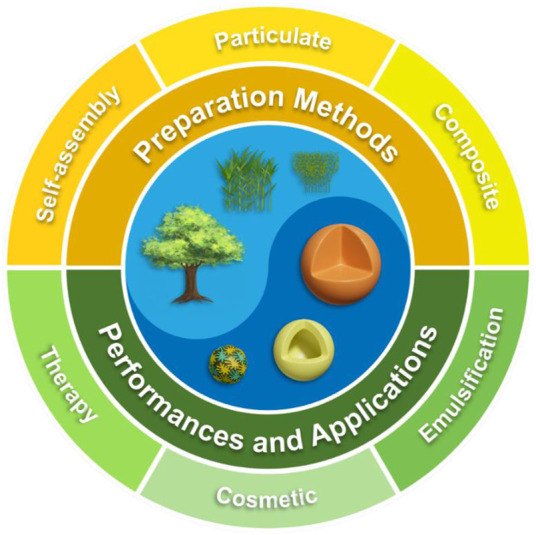
Illustration of some lignin colloids structured with several preparation methods toward significant performances and applications.

Currently, most studies suggest that π-π stacking and hydrogen bonding between lignin molecules lead to the formation of nanospheres. Although several researchers have focused on the formation mechanism of LNCs through molecular simulations or experimental approaches, little progress has been made (Chee et al., 2020; Pylypchuk et al., 2020). In this paper, the assembly of lignin in single or mixed solvents, and the preparation methods of composite lignin particles were reviewed. The aggregation and self-assembly of lignin as well as the synthetic methods of particulate materials were summarized. The applications of hybrid LNCs composited with these functional substances in the fields of therapy, cosmetics, and emulsification were prospected (Figure 1). It is hoped that our review will contribute to the research on lignin self-assembly and the high value utilization of LNCs.

## 2 Lignin

Lignin is widespread in plants and forms the plant skeleton together with cellulose and hemicellulose as the main components through partial covalent bonds and intermolecular forces. Lignin makes an important contribution to plant survival by providing not only excellent mechanical properties but also good resistance to disease, insects, flooding, cold and UV (Wang et al., 2020). As an important component of biomass resources, lignin is a valuable resource endowed by nature to mankind. As an important non-fossil carbon source, it is second only to cellulose in natural reserves (Mishra and Ekielski, 2019) and is also a major source of aromatic biopolymers in nature (Kai et al., 2016).

### 2.1 The concept and structural composition of lignin

**FIGURE 2:**
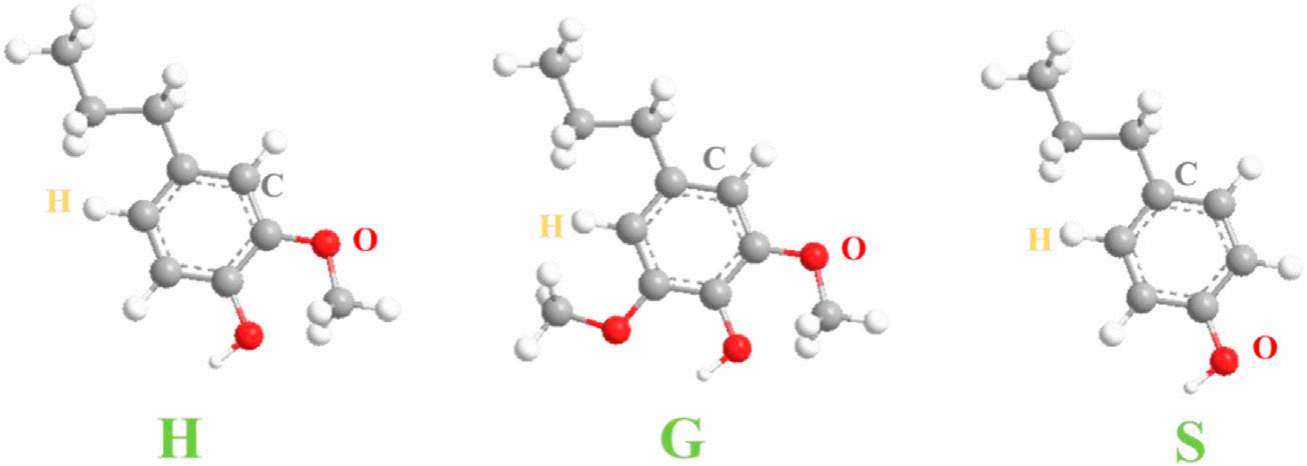
Structural units of lignin macromolecules.

The lignification of plant cell walls is a polymerization process in which lignin monomers are converted into lignin macromolecules through free radical polymerization and oxidative coupling reaction. The initial stage of lignification is started by dehydrodimerization of the same monolignol molecule or mutual dimerization of two different monolignols. The formed dimer further undergoes cross-linking coupling reaction with the monolignol and oligomer and is connected together by an ester bond, an ether bond, a carbon-carbon bond and the like to form a lignin macromolecule (Marita et al., 2016). Lignin is an irregular aromatic polymer composed of p-hydroxyphenyl (H), guaiacyl (G) and syringoyl (S)-type phenylpropane units (Iravani and Varma, 2020) (Figure 2). Among them, β-O-4 and α-O-4 bonds (accounting for about 50%) were the main linkage of monomers, and they were also linked by β-5, β-1, and five to five, etc. The structure of lignin is relatively complex, and there are many functional groups such as methoxy group (-CH_3_), hydroxyl group (-OH), and carbonyl group (-C=O). Due to the existence of these functional groups, lignin has a variety of chemical properties and high reaction activity, which plays an irreplaceable role in the high-value utilization of lignin (Wijaya et al., 2021).

Methoxy group is one of the representative functional groups mentioned above. Lignin methoxy is relatively stable and can only be broken and shed by strong oxidizing agents or cleaved to form methanol under high temperature and pressure reaction conditions. In addition to the above-mentioned cleavage process, the modification process is more specific and the hydroxyl group is usually the most representative and the most modified functional site. The hydroxyl group is one of the important functional groups of lignin and exists in two forms in lignin, the aliphatic alcohol hydroxyl group present on the side chain of the benzene ring in lignin and the phenolic hydroxyl group on the benzene ring of lignin. These hydroxyl groups can either be attached to other alkyl and aryl groups in the form of ethers or exist as free hydroxyl groups (Thakur et al., 2014). Lignin has strong intramolecular and intermolecular hydrogen bonds also both due to the action of hydroxyl groups. The carbonyl group is on the side chain of the benzene ring, one that is conjugated to the benzene ring and one that is non-conjugated. The double bond is thought to be formed by the dehydrogenative polymerization of lignin from cinnamyl alcohol, so that the unsaturated side chain is retained as a terminal group.

### 2.2 Sources of industrial lignin

The lignin obtained by treatment and separation using different industrial methods is industrial lignin. Depending on the treatment and separation method, the common industrial lignin is mainly classified as alkaline lignin (AL), lignin sulfonate, enzymatic lignin and organic solvent lignin. Depending on the separation process, the structure and the type and content of the active groups of lignin vary considerably. This review will also focus on common industrial lignin and will focus more on the value of their industrial derivatives.

AL is derived from the lignin in the “black liquor” of alkali pulping. In the pulping process, high temperatures and strong alkali conditions introduce a large number of phenolic hydroxyl groups to the lignin in the raw material, which are dissolved in a strong alkaline solution after being turned into phenol-oxygen ions (Yan et al., 2010), so that the lignin can be effectively separated and removed as the cellulose and hemicellulose in the raw material are not dissolved. Due to the violent reaction conditions of this process, the lignin is turned into phenoxy ions while the ether bonds between the basic units are broken, and the large lignin molecules are degraded into smaller molecules, so the molecular weight of AL is generally smaller. AL is highly hydrophobic and insoluble in water but can be dissolved in alkaline aqueous solutions. Due to its poor water solubility and low surface activity, AL is generally surface modified to improve its water solubility or reactivity before it can be used.

Lignosulphonate is a by-product of sulphite pulping. In contrast to alkali pulping, sulphite is added to the pulp without the use of strong alkali. The lignin in the raw materials reacts at high temperature. At the same time of introducing sulfonic acid group, the connection bonds between the basic structural units are broken to produce water-soluble lignosulfonate (Ouyang et al., 2006), which is separated by dissolution in water. Since lignin sulfonates have both a hydrophobic benzene ring and alkyl chain structure as well as hydrophilic functional groups such as carboxyl and phenolic hydroxyl groups, they can act as an amphiphilic surfactant (Li et al., 2011).

Enzymatic lignin is a by-product of the biorefinery ethanol industry and is derived from the residue of cellulose enzymatic digestion. Because bioenzymatic digestion selectively opens the linkage bonds between lignin and carbohydrates under a milder condition, the separation of lignin is achieved. The lignin produced by this process is therefore able to retain its original macromolecular structure to the greatest extent possible (Jin et al., 2010). Since enzymatic lignin is of higher quality, cleaner and has greater structural retention, it is more often used as a feedstock to study lignin structure, pyrolysis properties and the utilization of pyrolysis products (Rui et al., 2010).

Organic solvent lignin is extracted from plants using solvents such as methanol, formic acid, acetone, ethanol and ethanol-water (Scholze and Meier, 2001). The separation efficiency of organic solvent extraction is high and the resulting lignin is pure, low in molecular weight and hydrophobic, with potential for high value added applications (Lora and Glasser, 2002).

## 3 Self-assembly of lignin

In recent years, research on the comprehensive utilization of lignin has received more and more attention from scholars. The key scientific problem in solving the comprehensive utilization of lignin lies in, firstly, clarifying the molecular structures of lignin macromolecules at various levels; secondly, sorting out the interactions between these molecular structures; and most importantly, clarifying the orderly assembly morphology between the molecules at various levels. Since existing technologies cannot precisely achieve the selective disassembly of lignin macromolecules at all levels, using the structural features of the assembled levels to directly design and manufacture advanced materials will become the most convenient means. Self-assembly is the process of forming an organized structure or pattern from the presence of disordered subunits, under the condition that it is independent of external factors and driven only by internal forces and interactions occurring within the system (Mishra and Ekielski, 2019). Lignin forms aggregates in organic solvents based on π-π interactions of aromatic groups, from inside to outside, by means of layer-by-layer self-assembly (Xiong et al., 2017b). Self-assembly of lignin is a complex thermodynamic-dependent and kinetic-dependent process, which is attributed to the increase in attractive interactions between lignin molecules. In natural and synthetic systems, stacking of aromatic rings is a very common non-covalent interaction that controls the structure and assembly of macromolecules (Salentinig and Schubert, 2017). Due to the diversity of lignin functional groups, the forces between lignin molecules are complex, where each of the long-range interactions (e.g., van der Waals forces, electrostatic forces and hydrophobic interactions) and short-range interactions (e.g., hydrogen bonding and π-π interactions) combine with each other. Many researchers have studied the self-assembly process of lignin and qualitatively demonstrated that one or both of these intermolecular forces are the driving forces in the self-assembly process (Wang et al., 2022).

### 3.1 Self-assembly of lignin in single solvent

Lignin macromolecules are complex biomolecules obtained by the polymerization of different species of units. In solvents, lignin macromolecules that have dissociated some of their weak interactions can further achieve new aggregations through new interactions in good solvents, and the different aggregation behaviors bounded by different solvents can influence the formation of different assemblies of lignin macromolecules. This is the focus of molecular science, polymer science and even colloid science. So far, the deconstruction and reconfiguration of lignin macromolecules has been a difficult task, and the “holistic orderly utilization of fully soluble/dispersed units” is expected to quickly solve the problem of structural utilization of lignin. Currently, lignin and its derivatives are commonly used in industry, including AL, sodium lignin sulfonate and enzymatic lignin. In the following we will focus on their assembly behavior in different solvents, providing an important approach to the “holistic and orderly utilization of fully soluble/dispersible lignin-macromolecules units”.

AL can form two-stage assembly in the solvent: one is the polymer chain molecular aggregation caused by van der Waals attraction; the other is the π-π aggregation of aromatic groups in lignin due to the bond-free orbital interaction (π-π interaction) (McRae and Kasha, 1958). The π-π aggregation was first named based on the relative positions of the aggregation absorption band and the molecular absorption band (m-band) (Dash et al., 2011). Molecular aggregation is not accompanied by energy transfer, while π-π aggregation is usually accompanied by energy splitting. Therefore, π-π aggregation of aromatic compounds does not necessarily result in molecular aggregation, and *vice versa*.

**FIGURE 3:**
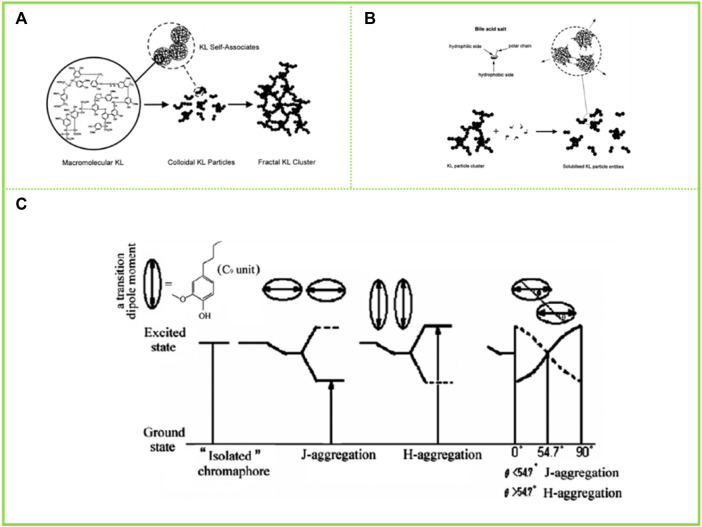
**(A)** schematic representation of the modes of aggregation in kraft lignin (KL) systems starting from macromolecular KL (ref 49) and finally reaching fractal KL clusters (Norgren et al., 2002) **(B)** A schematic illustration, showing the bile salt–KL interaction (Magnus Norgren 2001) **(C)** Schematic representation of the arrangement for H and J aggregation and their different energy levels (Deng et al., 2011). **(A)** Copyright 2002 American Chemical Society **(B)** Copyright 2001 Elsevier **(C)** Copyright 2011 American Chemical Society.

Lindström (1982) proposed that carboxyl group played an important role in the association and the thermally irreversible property of association in aqueous AL solution, and further proposed that AL aggregates were formed by hydrogen bonds between carboxyl group and various ether oxygen and hydroxyl groups. However, Sarkanen et al. (1981) reported in their study that the aggregation process of AL is limited by stoichiometry, with each AL aggregate having a site that is complementary to only one type of component, respectively. Thus, they proposed that the aggregation of AL is controlled by π-π interactions between benzenes. With the increase of modern analytical and testing methods, more high-end instruments and equipment have realized the deconstruction of the assembled morphology of lignin macromolecules. Norgren et al. (2001); Norgren et al., 2002) studied the aggregation behavior of AL in alkaline aqueous solution using light scattering and turbidimeter. It was found that AL initially existed in solution as colloidal macromolecules, and when the solution conditions changed (e.g., addition of electrolytes) the AL macromolecules first cross-linked into colloidal particles, then the number of colloidal particles increased and the particle size increased, and finally these colloidal particles formed clusters and aggregates, indicating that the electrolyte in solution had a significant effect on the aggregation behavior of AL (Figures 3A,B). Meanwhile, Kubo and Kadla, (2005) investigated the formation of lignin hydrogen bonds by infrared spectroscopy using lignin model compounds, demonstrating that hydrogen bonds are one of the driving forces for lignin aggregation.

Apart from studies on the assembly behavior of AL in aqueous solvents, tetrahydrofuran (THF) remains one of the most widely utilized solvents to study the assembly behavior of lignin. Deng et al. (2011), in his study of the aggregation behavior of AL in THF solution, found that AL forms two different aggregation modes in THF solution, π-π aggregation and molecular aggregation (Figure 3C). AL tends to form molecular aggregates and π-π interactions of aromatic rings are responsible for the lignin association phenomenon in organic solvents. The π-π interactions of aromatic rings lead to a pattern through which AL can aggregate, such as sandwich-type arrangements and head-to-tail arrangements, similar to the well-known H and J aggregation in organic chromophores. Later studies found that AL coexisted in THF in the form of single molecule and aggregation. Because inter- and intra-molecular aggregation due to hydrogen bonding and π-π interactions is stronger for AL, it exists in solution as a single molecule and as an aggregate, and the molecules are more curled. After acetylation the inter- and intra-molecular aggregation in THF is reduced and the resulting acetylated AL is more often present as a single molecule in THF, with more extended molecules and a larger hydrodynamic radius.

### 3.2 Self-assembly of lignin in mixed solvents

In the lignin self-assembly process, two or even more solvents can be mixed: a good solvent, named Solvent I, which interacts strongly with lignin and dissolves easily, and an anti-solvent, named Solvent II, which interacts weakly with lignin and does not dissolve well. The dissolution of lignin in solvent I and the aggregation in solvent-II are the two key steps. By adjusting the properties and ratios of solvent I and solvent II, the dissolution and aggregation processes can be precisely controlled (Wang et al., 2020). Lignin is soluble in organic solvents such as THF (Contreras et al., 2008; Qian et al., 2014a; Qian et al., 2015a), acetone (Yearla and Padmasree, 2015; Beisl et al., 2020; Zhang et al., 2022), ethanol (Rao et al., 2017; Tang et al., 2017; Li et al., 2018; Del Buono et al., 2022), ethylene glycol (Yang et al., 2018), and acetonitrile (Xu T. et al., 2022) in which aggregation follows a nucleation-growth mechanism. Nucleation is initiated by the precipitation of large lignin fragments as precursors to critical nucleation. The growth process gradually turns into larger particles through collision-driven particle aggregation and fusion.

#### 3.2.1 Self-assembly of lignin in THF-water

**FIGURE 4:**
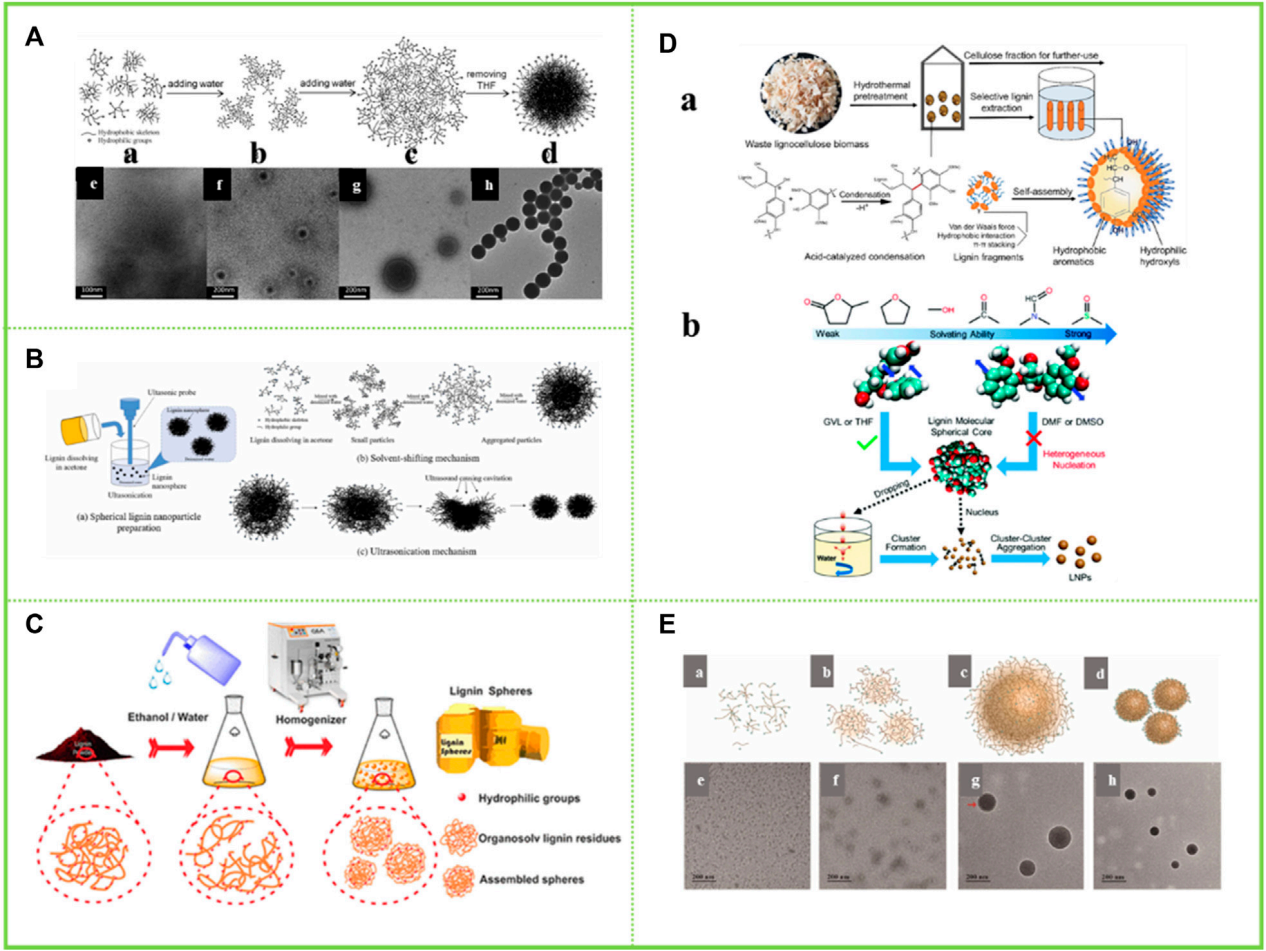
**(A)** Schematic representation of the colloid formation process of the acetylated lignin (ACL) in a THF–H_2_O medium and TEM images of the samples obtained from the dispersions with different water contents (Qian et al., 2014b) **(B)** Schematic of (a) spherical lignin nanoparticle preparation; (b) self-assembly mechanism and (c) ultrasonic mechanism (Rao et al., 2017) **(C)** Schematic diagram shows the aggregation and regeneration of the OLR-based submicron spheres (Rao et al., 2017) **(D)** Lignin condensation facilitates its solution based self-assembly for the production of LNCs with spherical structure (Chen et al., 2020), formation mechanism of LNCs from different solvents (Chen et al., 2022) **(E)** Proposed schematic illustration of colloid formation process of the lignin in DES (Zhang et al., 2022). **(A)** Copyright 2014 Royal Society of Chemistry **(B,C)** Copyright 2017 Elsevier **(D)** Copyright 2020 Elsevier, 2022 Royal Society of Chemistry **(E)** Copyright 2022 Elsevier.

Tetrahydrofuran is a polar non-protonic ether with good solubility for lignin. Qian et al. (2014a); Qian et al. (2014b) dissolves the acetylated AL in THF. Since the basic unit of lignin is phenylpropane, and it is rich in π-π bonds, π-π interaction between lignin molecules and π-π interaction between aromatic nuclei and other conjugated structures play an important role in the aggregation of lignin molecules. The lignin colloidal particles are then obtained by adding water to form a hydrophobic effect. This enables the simple acquisition of LNCs without any chemical modification (Figure 4A). This is because when a lignin dissolution solution is added to water, the stretched lignin molecules tend to fold into a stacked structure, thus aggregating to obtain lignin colloidal spheres (LCS) (Spruce milled wood lignin: linear, branched or cross-linked) (Chen et al., 2022). This theory provides an important theoretical basis for the self-assembly behavior of lignin in tetrahydrofuran-water solvent and has important guiding significance for the subsequent research. Smith et al. (2016) applied all-atom molecular dynamics simulations (MD) to investigate the structure of lignin in a THF-H_2_O co-solvent environment. It was found that pure H_2_O was a poor solvent for lignin, while THF-H_2_O was a “good solvent” for lignin. Lignin, though largely hydrophobic, does have the ability to form lignin-H_2_O hydrogen bonds with water. As with lignin-lignin hydrogen bonds, the presence of THF reduces the propensity for lignin-H_2_O hydrogen bond formation. Xiong et al. (2017a) prepared lignin hollow nanospheres with a single pore in a THF-H_2_O solution. The most likely explanation for the formation of lignin hollow nanostructures during the assembly process is that the driving force during the assembly process comes from the small amounts of impurities present in the THF and it is the level of THF purity that leads to the phase separation of THF from water. Because the formation process of the hollow spheres shows that the lignin is completely dissolved in THF, by adding water, the more hydrophobic lignin molecules form a film at the interface between the two phases of water and THF, resulting in the water being encapsulated. The increase in water content causes the pressure gradient inside and outside the membrane to increase, resulting in an opposite rotation. The lignin tetrahydrofuran solution is encapsulated by the membrane. As the water content increases further, more and more water molecules penetrate the membrane, resulting in more and more lignin molecules collecting on the inner surface of the membrane in a layer-by-layer self-assembly based on π-π interactions. Eventually, the thinner side of the film breaks off and a lignin hollow nanostructure is obtained. Xiong et al. (2020) carried out a further study. They found that the food additive butylhydroxytoluene (BHT) could modulate the solid/hollow structure of lignin nanospheres. This is because the lignin nanospheres obtained in tetrahydrofuran (THF) with a BHT concentration greater than 0.3 mg/ml showed a hollow structure, while those without the addition of BHT showed a solid structure. Chen et al. (2022) also elaborated the dissolution behavior of lignin in THF-H_2_O. In solvents with certain solvating power such as THF, the lignin molecules maintained intramolecular accumulation, and the intramolecular aromatic ring interaction of guaifenesin guaiacylglycerol-beta-guaiacyl ether (GGE) was similar to that in water, resulting in the formation of spherical seeds during heterogeneous nucleation. When a high concentration of lignin solution was put into water, these nuclei form clusters and then further aggregated into LNCs.

#### 3.2.2 Self-assembly of lignin in acetone-water

The preparation of lignin colloidal nanoparticles can likewise be achieved with acetone as one of the good solvents for lignin. In systems where pure acetone is completely miscible with water, lignin hollow nanoparticles can be obtained. The reason for this is that the assembly of vesicles during the addition of water is a consequence of the hydrophobic effect and maximises the π-π interaction between the aromatic hydrophobic parts of the lignin. Due to the amphiphilic nature of lignin, the formation of vesicle-like structures increases the density of intermolecular interactions between the hydrophilic part of lignin and water on the inner and outer surfaces (Trevisan and Rezende, 2020). During the preparation of samples for electron microscopy analysis, hollow spheres were formed as water evaporated. Winotapun et al. (2022) and others dissolved the isolated lignin in acetone and further added deionised water and found that hydrophobic molecules on the polymer chains were the first to assemble and aggregate into nuclei and that phase separation during solvent exchange promoted the formation of lignin colloids (Figure 4B). In other words, the lignin molecules are redistributed in the acetone/water solvent mixture according to their polarity and hydrophobicity, as they are less soluble in water. These structures can only be degraded by the addition of a solvent that wins energy, which has a higher interaction energy with π-π and hydrogen bonds than with the solvent. Therefore, it can be summarized that the core of colloidal lignin is composed of the most hydrophobic part with high molecular weight and the part with less polarity. When excess water is added to the lignin-acetone solution, most of the polar molecules dissolve in the acetone/water solvent mixture for as long as possible and when the acetone evaporates, they adsorb on the surface of the lignin nanoparticle core to form a low-density layer, which in turn forms lignin colloidal nanoparticles (Pylypchuk et al., 2020). Beisl et al. (2020) dissolved wheat straw in acetone and induced self-assembly by adding water and evaporation to obtain LNCs. This particle existed as a lignin agglomerate with a size of around 300–400 nm.

#### 3.2.3 Self-assembly of lignin in ethanol-water

Given the good solubility of sodium lignosulfonate in ethanol, Tang et al. (2017) investigated the homogeneous colloidal lignin obtained by self-assembly in ethanol solution after mixing aqueous sodium lignosulfonate (NaLS) and cetyltrimethylammonium bromide (CTAB). The results show that the polymerization of NaLS can be divided into two types, π-π aggregation of aromatic groups in NaLS molecules caused by π-π interactions (non-bonded orbital interactions) and molecular aggregation of polymer chains caused by van der Waals forces. π-π aggregation is usually accompanied by energy splitting and molecular aggregation is not accompanied by energy transfer. The emergence of this study has provided important ideas for understanding the self-assembly behavior of lignin in green organic solvents and has made an important contribution to the high-value application of lignin. Rao et al. (2017) and Pang et al. (2020) chose different lignin raw materials, i.e. organic solvent lignin and enzymatic lignin, respectively, to obtain lignin micro/nano-particles with different phenolic hydroxyl content and different molecular weights in aqueous ethanol solutions (Figure 4C). In ethanol-water solutions, the hydrophilic part of the lignin molecule promotes its affinity for ethanol, while the hydrophobic part promotes the formation of an electric double layer, forming spheres that are stably dispersed in ethanol-water solutions. Facilitated by van der Waals and π-π interactions, the hydrophobic region tends to aggregate to form the “yolk” of the colloidal sphere, while the regenerated dissociated lignin part forms the shell layer. At the same time, it is demonstrated that lignin spheres with different phenolic hydroxyl content and different molecular weights are obtained in different concentrations of ethanol-water solutions. As the concentration of ethanol decreases, the molecular weight of the lignin spheres formed increases and the hydroxyl content becomes lower. Thus, lignin heterogeneity has a strong influence in the formation of LNCs by lignin self-assembly.

#### 3.2.4 Self-assembly of lignin in dimethyl sulfoxide (DMSO)-water or N,N-dimethylformamide (DMF)-water

Chen et al. (2020) prepared LNCs in DMSO. When a counter-solvent (e.g., water) is introduced into the lignin solution, its hydrophobic part aggregates to form the inner core of the particle, while the hydrophilic parts of the phenolic and aliphatic hydroxyl groups form the shell layer (Figure 4D). In this process, the association of lignin fragments is influenced by intermolecular van der Waals forces, π-π stacking and hydrophobic interactions. In this work, it is hypothesized that the condensation of lignin between aromatics can enhance the driving force of the self-assembly process by providing more available hydrophobic anchors. Therefore, even if these lignin by-products were not hydrophobically modified, smaller particle size LNCs could be produced. However, a year after this idea was proposed, other researchers suggested the opposite. Chen et al. (2020) showed that nano-lignin could not be formed when DMSO and DMF were used as solvents for dissolving lignin (Figure 4D). This is because there is almost no light scattering from the suspensions of DMSO and DMF when solutions containing high concentrations of lignin are added drop by drop to water, respectively. At the same time, the suspensions were dialyzed against water to remove free organic solvent molecules and centrifugal force was applied to the dialyzed suspensions, and no precipitates were collected from the samples containing the DMSO and DMF solutions. When the solvent-free suspensions were air-dried and exposed to TEM, fibrous and flaky lignin particles were detected in DMF and DMSO, respectively. All these phenomena indicate that the use of DMF and DMSO as solvents for lignin does not produce homogeneous nanoparticles. This can be explained by the fact that DMSO and DMF are highly dissolved to lignin and the aromatic rings of lignin molecules are abstracted from the intramolecular stacking by solvent molecules such as DMSO, preventing lignin molecules from forming spherical cores and aggregating to form LNCs. Therefore, the preparation of homogeneous, stable and mass-produced LNCs by solvent exchange requires both solubilizing lignin and ensuring the formation of lignin spherical cores by solvent.

#### 3.2.5 Self-assembly of lignin in betaine-lactic acid deep eutectic solvent-water

Lignin is very soluble in DES and with the addition of progressively more water, some of the hydrophobic backbones of the lignin molecules tend to form localised clusters. Further increases in water content lead to the creation of more micelles, which then aggregate into clearly visible colloidal spheres. This phenomenon is similar to the micellization process of block copolymers, where the micelle core and micelle shell are formed from hydrophobic and hydrophilic blocks respectively. By adding excess water to disassemble the micellar structure, the presence of intermolecular interactions drives the lignin from an irregular conformation to a homogeneous spherical structure. In addition, the addition of water may have a significant effect on the dissociation and size of the DES clusters. This is because excess water breaks the hydrogen bonds in DES and gradually reduces the intermolecular interactions between DES and lignin, leading to a decrease in the solubility of lignin in DES (Zhang et al., 2022) (Figure 4E). The aqueous solution of p-toluenesulfonic acid (P-TsOH) of dried wheat straw flour lignin was sonicated at elevated temperature and then collected as water-insoluble solids by vacuum filtration, followed by repeated centrifugation to precipitate lignin and obtain lignin colloidal nanoparticles of uniform particle size (Yang et al., 2021).

#### 3.2.6 Self-assembly of lignin in dioxa-cyclohexane

**FIGURE 5:**
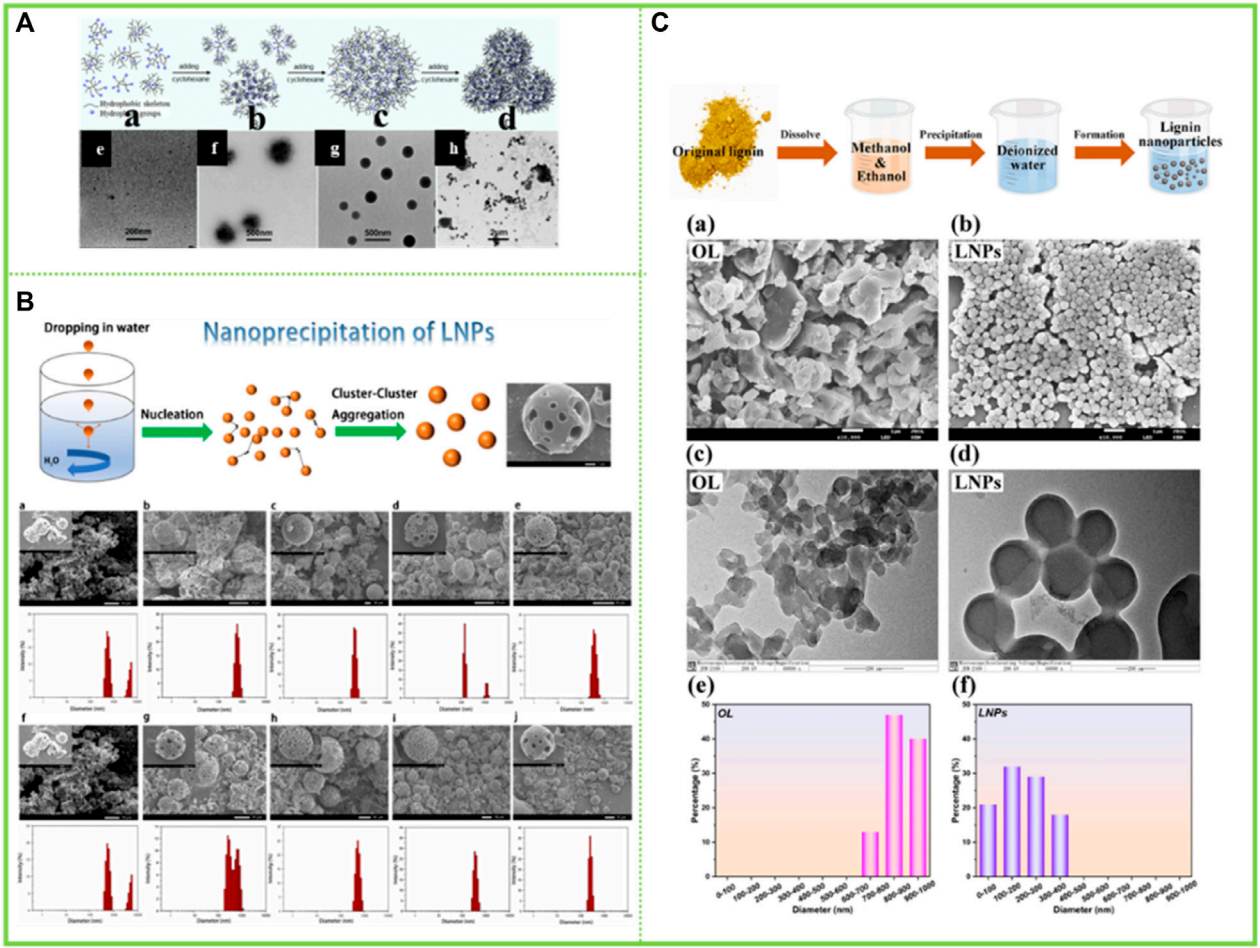
**(A)** Schematic representation of the formation process of LRM in dioxane−cyclohexane medium and TEM images of samples obtained from the dispersions with different cyclohexane contents (Qian et al., 2015a) **(B)** Lignin nanoparticle (LNP) formation mechanism during dropping nanoprecipitation, Scanning electron microscopy (SEM) images of LNCs (Yang et al., 2022) **(C)** Schematic diagram for the synthesis of LNCs, SEM images of (a) OL and (b) LNCs, TEM images of (c) OL and (d) LNCs, and nanoparticle size distribution of (e) OL and (f) LNCs (Du et al., 2022). **(A)** Copyright 2015 American Chemical Society **(B–C)** Copyright 2022 Elsevier.

In addition to the aforementioned reversed-phase assembly in organoleptic and water-resistant solvents, there have also been studies using two organic solvents to synergistically modulate the assembly behavior. Qian et al. (2015a) found that lignin in dioxane solution could generate lignin anti-micelles by the addition of cyclohexane, i.e. the formation of lignin anti-micelles consisting of a hydrophilic part inside and an exposed hydrophobic part (Figure 5A).

#### 3.2.7 Self-assembly of lignin in binary solvent-water

A few studies have also been carried out around more than two solvent systems. Yang et al. (2022) found that lignin was completely dissolved in the binary organic solvent mixture of dichloromethane and methanol when they dissolved lignin; However, when the above-mentioned lignin solution was added dropwise in deionized water as an anti-solvent, lignin molecules realized layer-by-layer self-assembly driven by π-π interaction between molecules, and finally lignin nanoparticles were obtained. Du et al. (2022) have similarly prepared lignin nanoparticles in a greener ethanol-methanol binary organic solvent mixture (Figures 5B,C).

#### 3.2.8 Self-assembly of various heterogeneous components of lignin

**FIGURE 6:**
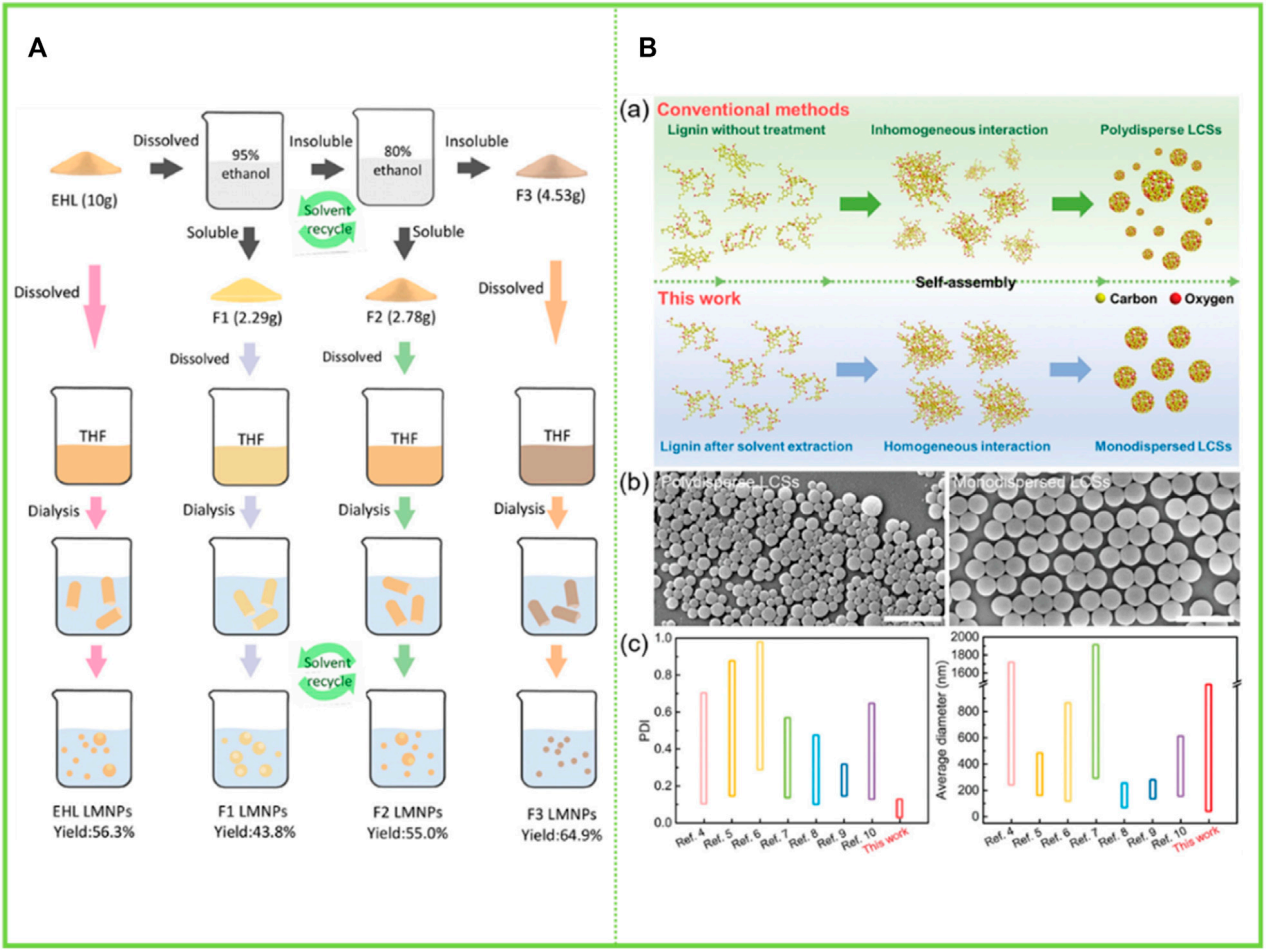
**(A)** Scheme for EHL fractionation by sequential dissolution in 95% and 80% ethanol/water solvents and the sequent lignin micro/nano-particles (LMNPs) preparation using anti-solvent precipitation method (self-assembly) (Pang et al., 2020) **(B)** Strategy for preparing monodispersed LCS with tailorable size (Wang et al., 2022). **(A)** Copyright 2020 American Chemical Society **(B)** Copyright 2022 Wiley.

Lignin is a biological macromolecule and it is difficult to precisely disentangle its detailed sub-assembly structure at all levels. However, in recent years, as the research on the comprehensive utilization of lignin is in full swing, the overall comprehensive utilization of lignin constituent units has become a hot topic. Pang et al. (2020) divided the enzymatic lignin into three components with gradually decreasing heterogeneity, namely a first component (F1) soluble in 95% ethanol solution, a second component (F2) soluble in 80% ethanol solution, and a residual lignin fraction (F3) insoluble in both 95% and 80% ethanol solution. The three graded groups were respectively dissolved in THF, and then THF was removed by adding water through a dialysis bag to obtain LNCs. The color of the obtained particles in the three groups gradually deepened, the total hydroxyl content gradually decreased, and the molecular weight gradually decreased. More importantly, the particles gradually become more uniform. F1 mainly formed incomplete spherical particles with relatively large size (450–650 nm), while F3 only formed small dense nanoparticles (about 50 nm) with relatively uniform size distribution. Two different particles with large hollow structure and small dense structure were prepared using enzymatic-hydrolysis lignin (EHL) and F2 as raw materials (Figure 6A). Wang et al. (2022) graded the enzymatic lignin so as to obtain homogeneous monodisperse LCS and improve the homogeneity of intermolecular forces: the soluble fraction of EHL in ethanol was dried to obtain F1, the insoluble fraction was washed and dried with water and mixed with acetone to obtain F2 and the insoluble fraction was F3. The F2 fraction is very homogeneous in particle size due to its high selectivity for solvents. This study successfully solved the problem of grossly inhomogeneous lignin composition, which greatly increases the commercial value of lignin. Similarly, the sieving of lignin macromolecules with similar structures through the selectivity of solvents to further reshape the uniform monodisperse system has become a hot research topic in recent years, with the most prominent research results in the preparation of homogeneous spheres by Xueqing Qiu’s team (Wang et al., 2022). They have proposed a new preparation method and produced homogeneous monodisperse lignin microspheres in large quantities with highly efficient and controllable structures and flexible size regulation across scales, which will bring new opportunities for the commercialisation of lignin (Figure 6B).

#### 3.2.9 Some considerable factors affecting the size of self-assembled colloidal spheres

Although there are many species of lignin and many types of lignin molecules, and it is difficult to standardise the assembly behavior of lignin molecules through absolute theory, some common features can be summarised and generalised. The molecular weight, original concentration, temperature, stirring rate and pH have important effects on the size of the colloidal spheres during the formation of lignin colloidal particles.

Molecular weight of lignin: The particle size of lignin nanospheres increases with the increase of molecular weight, and the polymer with higher molecular weight will have greater self-assembly tendency (Hasan and Fatehi, 2019). The polymer with higher molecular weight has low solubility and higher viscosity in organic solvents, resulting in high curing rate and increased particle size (Sameni et al., 2018).

Original concentration of lignin: During the formation of LNCs, a higher concentration of lignin in the system means that the nanoparticles have a higher probability of forming nuclei and more lignin is required for growth, and are also more likely to undergo aggregation, with the nucleation-growth mechanism occurring faster and the final LNCs formed having a larger average diameter (Lievonen et al., 2016).

Temperature: Adamcyk et al. (2022) investigated the effect of different temperatures on the formation process of LNCs and found that an increase in mixing temperature resulted in smaller particle size and a slight decrease in yield. This effect was strongest at the highest lignin concentrations.

Stirring rate: Li et al. (2022) controlled the stirring rate and deionised water addition rate in the preparation of LNCs. It was found that the particle size of lignin nanospheres increased and then decreased as the magnetic stirring rate and deionised water addition rate increased, probably because a higher stirring rate could improve the mixing performance of the aqueous and organic phases, but too low a stirring rate was not sufficient for complete mixing, and too high a stirring rate would destroy the harmony between the aqueous and organic phases.

pH values: The particle size of the lignin nanospheres was not significantly affected by pH during formation. However, after 7 days of preparation, the size of the nanospheres increased under low acid conditions, while under high alkaline conditions, the size of the nanospheres decreased significantly and the particles started to dissolve. This is because the change in pH leads to protonation or deprotonation of functional groups on the lignin coils, which controls the electrostatic interactions between the molecules (Li et al., 2022). At pH 5.6, above a pKa of 4.75 for carboxyl groups, most of these groups are deprotonated and negatively charged. The repulsive Coulomb forces between negatively charged substituents on the lignin chains may lead to the formation of loose polymer coils in micron-sized lignin particles. In contrast, at pH 2.0, the carboxyl groups are predominantly protonated and uncharged. The absence of repulsive charge interactions between subunits on lignin chains may lead to a more compact and dense arrangement of individual chains (Salentinig and Schubert, 2017).

## 4 Preparation of lignin composite particles

### 4.1 Lignin composited with organic matter

**FIGURE 7:**
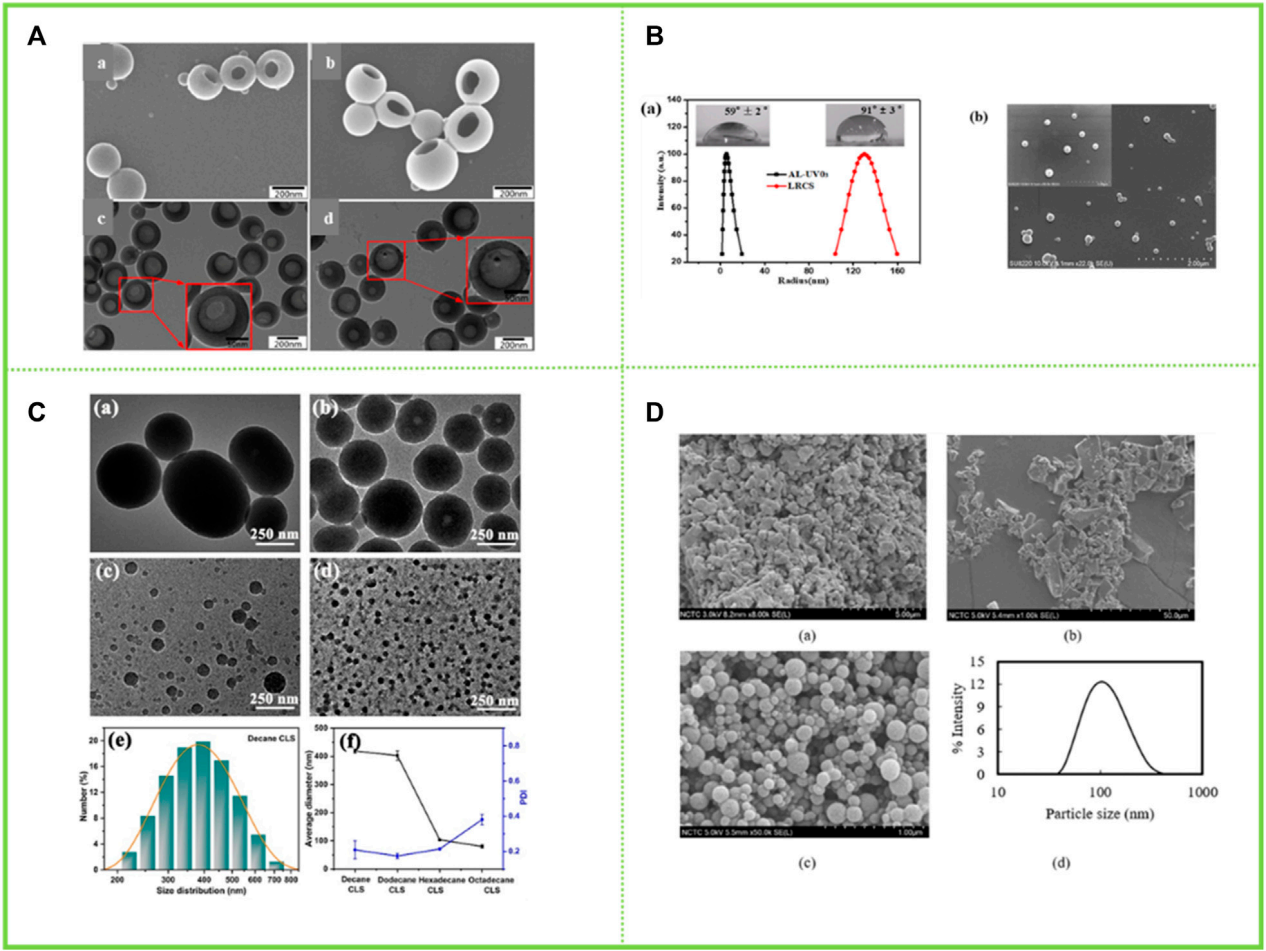
**(A)** SEM images of (a) lignin-based hollow nanoparticles (LHNPs), (b) CD-LHNPs, TEM images of (c) LHNPs, and (d) CD-LH (Zhou et al., 2019) **(B)** (a) Size distributions of AL-UV03 before and after reverse self-assembling, inset was the water contact angles of AL film and lignin reverse colloidal spheres film on glass surfaces; (b) SEM images of lignin reverse colloidal spheres (Wu et al., 2019) **(C)** TEM images of colloidal lignin sub-micro spheres with different alkyl chain (Li et al., 2020) **(D)** SEM images of (a) commercial softwood KL, (b) fractioned kraft softwood lignin, (c) LNCs, (d) particle size distribution of LNCs (Winotapun et al., 2022). **(A)** Copyright 2019 MDPI **(B)** Copyright 2020 Elsevier **(C)** Copyright 2019 American Chemical Society **(D)** Copyright 2022 Elsevier.

Zhou et al. (2019) firstly modified enzymolytic lignin EHL with aminopropyl triethoxysilane APTES, then grafted with β -cyclodextrin (β-CD), and then self-assembled the prepared small particles to form lignin hollow nanoparticles. The prepared nanoparticles were approximately 200 nm in size and their structure and properties remained stable in phosphate buffer at pH 7.4 for 6 d (Figure 7A). Wu et al. (2019) demethoxylated AL, grafted benzophenone, and reverse self-assembled in acetone solution to obtain lignin nanospheres (UV0) with a hydrophobic aromatic backbone distributed outside and in contact with non-polar acetone, while hydrophilic groups were arranged inside to form a polar core (Figure 7B). Li et al. (2020) synthesized four alkylated corncob lignin with similar chemical properties but different interfacial interactions by modifying them with four n-alkanes in order to modulate the amphiphilic nature of corncob lignin sub-micro spheres (Figure 7C). The results showed that the hydrophobic interaction between the n-alkanes and the aromatic backbone promoted the aggregation of corncob lignin. By modulating the hydrophobic and hydrophilic interactions, the formation of corncob lignin submicron microspheres with tunable surface properties can be successfully promoted. The greater the hydrophobic interactions, the longer the alkyl chain, the smaller the size of the corncob lignin sub-micro spheres and the better the stability. Winotapun (Winotapun et al., 2022) self-assembled lignin in the organic solvent acetone to obtain lignin nanosphere particles and then composited with chitosan. And this material was dehydrated at 40°C for 48 h to produce chitosan/lignin nanocomposites (Figure 7D).

### 4.2 Lignin composited with inorganic substances

**FIGURE 8:**
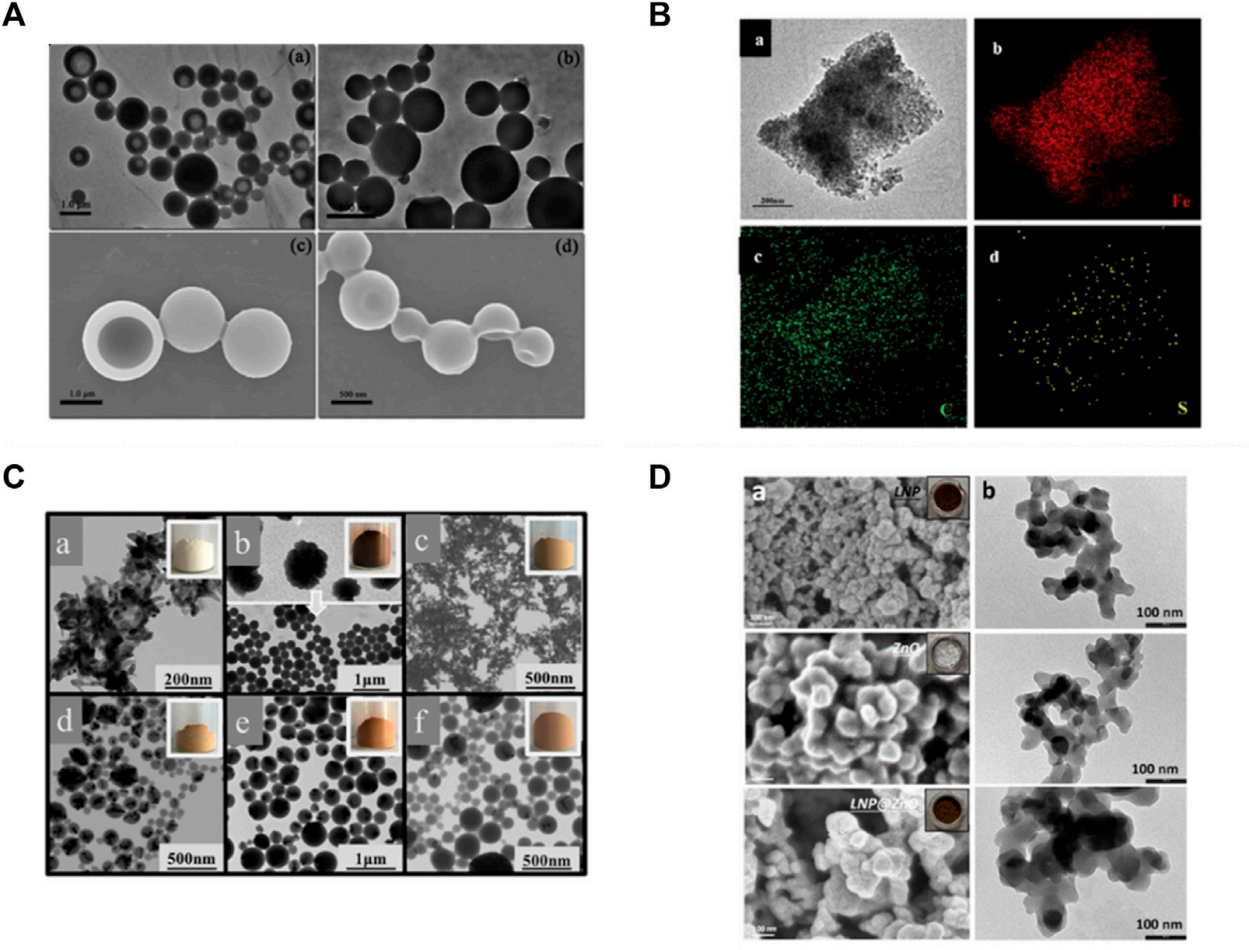
**(A)** TEM and SEM images of lignin hollow microspheres (LHM) prepared with unmodified larch (a,c) and poplar (b and d) lignin (Li et al., 2016) **(B)** TEM image of MLS25% and EDS mapping patterns of MLS25% (Geng et al., 2019) **(C)** TEM and appearance images of (a) TiO_2_, (b) QAL and its colloidal spheres, (c) LCS@TiO_2_-1, (d) LCS@TiO_2_-2, (e) LCS@TiO_2_-3 and (f) LCS@TiO_2_-4 (Li et al., 2019) **(D)** FESEM (a) and TEM (b) images of LNP, ZnO, and ZnO@LNP (the inset images show the color change due to the formation of lignin-derived ZnO) (Del Buono et al., 2022). **(A)** Copyright 2016 American Chemical Society **(B)** Copyright 2019 Elsevier **(C)** Copyright 2019 American Chemical Society **(D)** Copyright 2022 MDPI.

**FIGURE 9:**
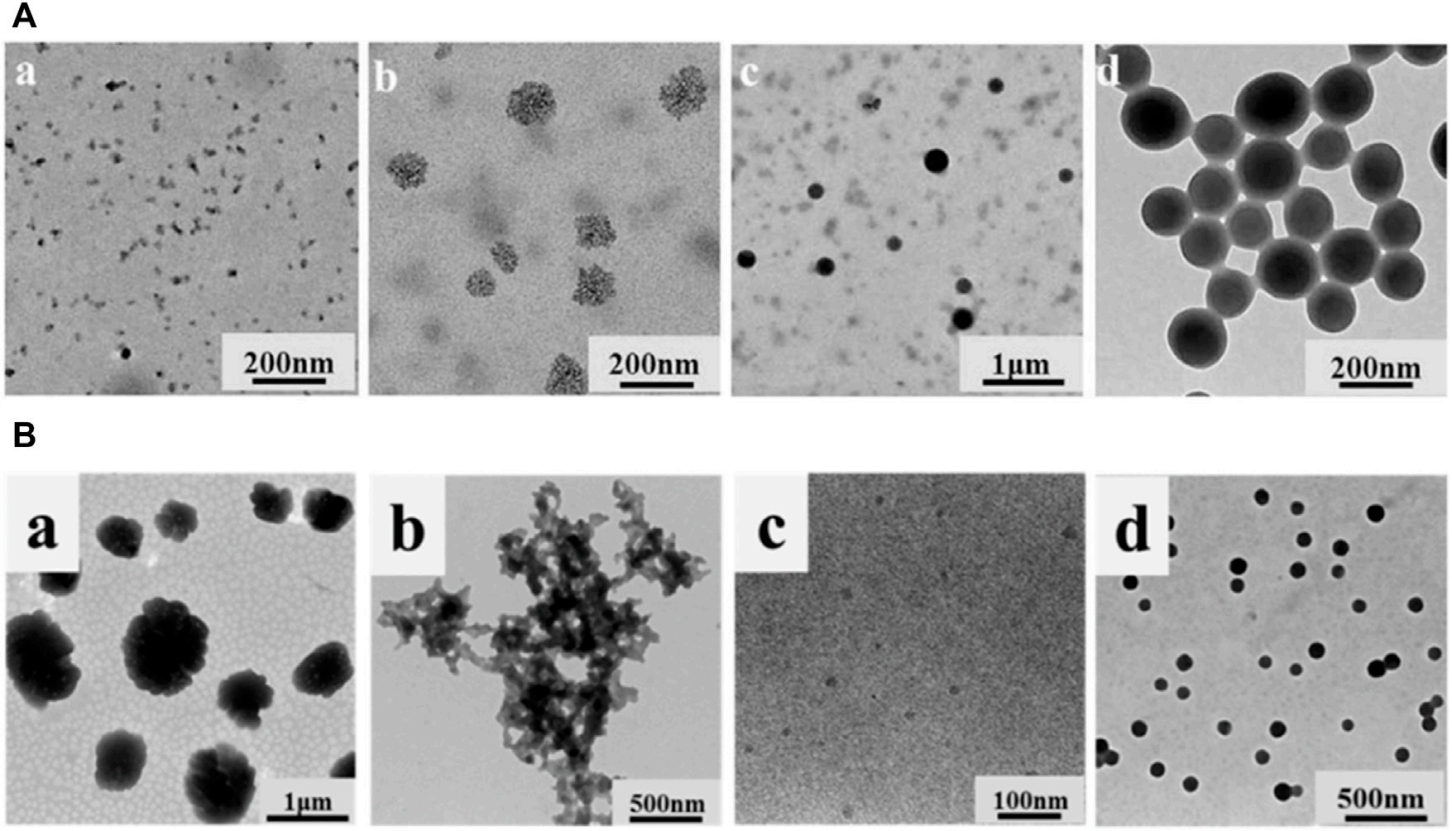
**(A)** TEM images of the samples at different water contents (Li et al., 2017) **(B)** TEM images of (a) QAL, (b) m(SDBS)/(QAL) = 0.6 complex, (c) m(SDBS)/m(QAL) = 0.6 complex solution in ethanol, and (d) colloidal spheres from the m(SDBS)/m(QAL) = 0.6 complex (Li et al., 2018). **(A)** Copyright 2017 Elsevier **(B)** Copyright 2018 American Chemical Society.

Li et al. (2016) adsorbed Fe_3_O_4_ nanoparticles with a diameter of 11 nm onto hollow lignin microspheres with a diameter of 500–1700 nm through the electrostatic interaction between electron-rich oxygen and hydroxyl groups of lignin, thus preparing the rough surface of magnetic lignin spheres (MLS). The holes in the lignin spheres provided a fixed site for Fe_3_O_4_ nanoparticles, and the magnetization saturation strength of the obtained lignin hybrid particles was 22.7 emu·g^−1^ (Figure 8A). Geng et al. (2019) used a one-step method to add FeSO_4_·7H_2_O and FeCl_3_·6H_2_O to an aqueous solution of sodium lignosulfonate, followed by the addition of NaOH and sonication to prepare a magnetic lignosulfonate consisting of uniformly distributed Fe_3_O_4_ nanoparticles in nematic form (Figure 8B). Li et al. (2019) compounded quaternised lignin (QAL) with sodium dodecyl benzene sulfonate (SDBS) to form QAL/SDBS complexes, which were prepared as LCS, and self-assembled and encapsulated TiO_2_ to form LCS@TiO_2_ composite microspheres (Figure 8C). TiO_2_ nanoparticles are rod-shaped and heavily agglomerated. QAL formed microscale agglomerates in lignin through hydrogen bonding and π-π interactions. QAL and SDBS in ethanol-water mixture can self-assemble into regular LCS. When the QAL/SDBS complex was less, the QAL/SDBS complex formed only a thin layer on the TiO_2_ surface. As the QAL/SDBS complex increased, the QAL/SDBS complex formed colloidal spheres and the TiO_2_ particles were effectively encapsulated in the LCS, as seen in Figures e-f. The black and translucent portions of the micrographs represent the TiO_2_ particles and the QAL/SDBS complex, respectively. The particle size of LCS@TiO_2_ composite microspheres ranged from 100 to 300 nm. Del Buono et al. (2022) added zinc acetate under the protection of argon in sodium bicarbonate and calcined at 350°C for 1 h to obtain zinc oxide-lignin nanocomposite particles (Figure 9D). Tian et al. (2017) used lignin as a structure-directed reagent and sulfuric acid as a precipitation reagent to prepare lignin/silica nanocomposite particles with good structural morphology, dispersion and adsorption properties.

### 4.3 Lignin composited with polymers

Li et al. compounded SDBS onto the surface of QAL to form a complex through electrostatic interaction and hydrophobic interaction, and then selected ibuprofen (IBU) (Li et al., 2017) and photosensitive pesticide abamectin (AVM) (Li et al., 2018) as model drugs, and incorporated them into the micelles to obtain functionalized lignin colloidal particles (Figure 9). After the SDBS/QAL complex was obtained, AVM was dissolved in ethanol solution, and a certain amount of SDBS/QAL complex was added for rapid mixing by peristalsis to obtain drug-loaded colloidal spheres. And finally collect by adopting a centrifugal freeze dry method. Finally, they were collected by centrifugal freeze-drying.

## 5 Applications of lignin colloids

### 5.1 Therapy

LNCs are characterized by low cytotoxicity and biocompatibility and can be used in tissue engineering, bio-based drug carriers and drug slow release. The colloidal spheres obtained by simple self-assembly behavior of lignin have a hydrophobic core and a hydrophilic shell, allowing for the encapsulation of hydrophobic drugs, and lignin has a three-dimensional network structure to control the release of drugs. In addition to this, LCS have excellent UV protection, which will also improve the resistance of the drug to photolysis. These advantages have led to the important development of lignin for drug encapsulation and release applications. Ibuprofen (IBU) is a well-known NSAID widely used for the treatment of various musculoskeletal and pain disorders. IBU is a good candidate for the development of oral controlled release formulations (Pourjavadi and Barzegar, 2009). Li et al. (2017) chose IBU as a model drug and added sodium dodecylbenzene sulfonate (SDBS) to complex it on the surface of QAL *via* electrostatic and hydrophobic interactions and incorporated IBU into micelles, thus achieving good compatibility of the micelles with IBU. The following year, Li et al. (2018) formed LCS of SDBS with QAL complexes in an ethanol/water mixture and encapsulated the photosensitizing drug avermectin (AVM), and the resulting product showed excellent controlled release, encapsulating up to 63.19% while maintaining up to 85.31% of the capacity release.

**FIGURE 10:**
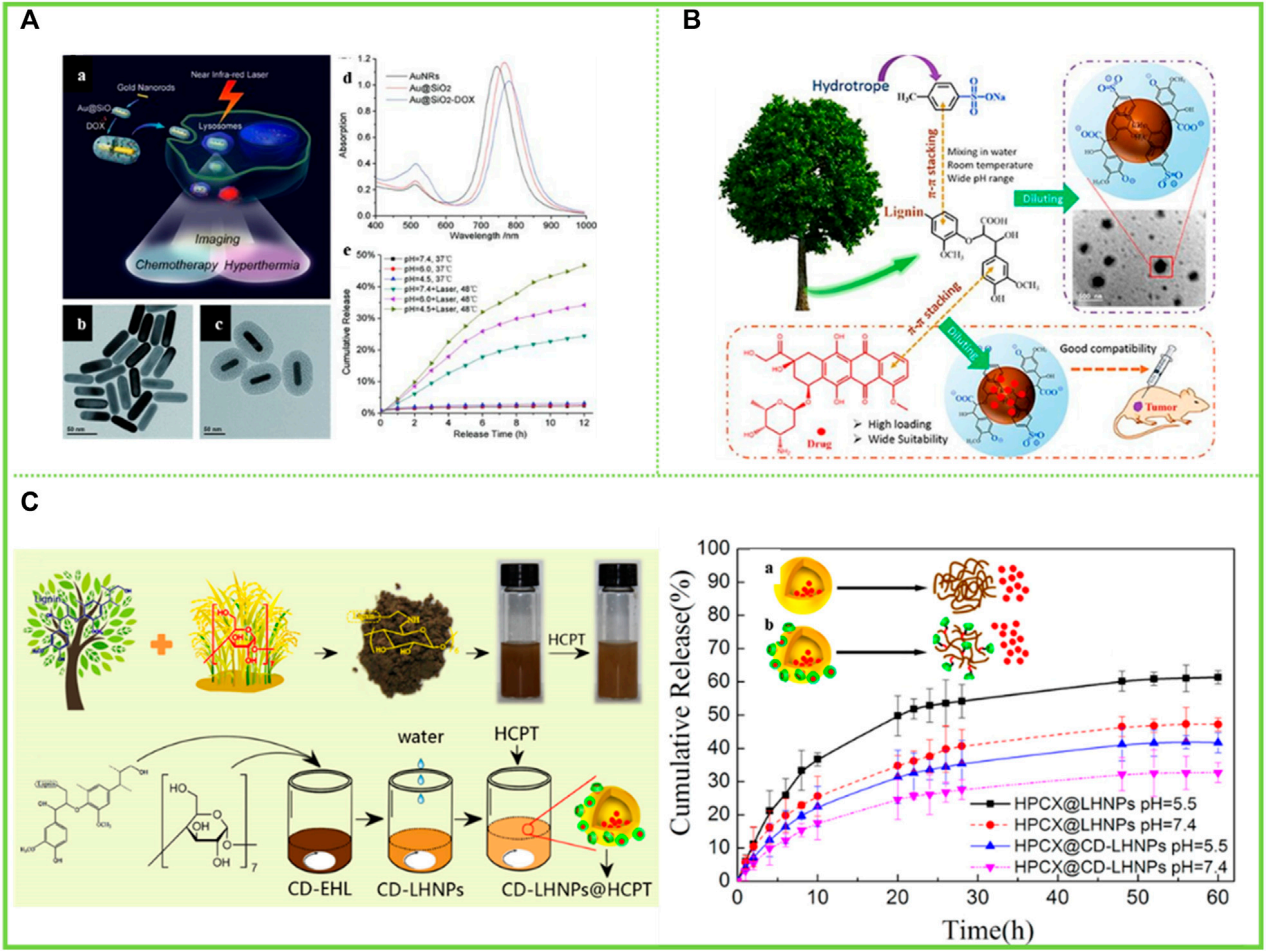
**(A)** (a) Schematic illustration of mesoporous silica-coated gold nanorods (Au@SiO_2_) as a novel multifunctional theranostic platform for cancer treatment. TEM images of (b) AuNRs and (c) Au@SiO_2_, (d) extinction spectra of AuNRs, Au@SiO_2_, and Au@SiO_2_–DOX, and (e) DOX release profiles from Au@SiO_2_–DOX with and without NIR laser irradiation at different pHs (Zhang et al., 2012) **(B)** Synthesis and application of LNCs (Chen et al., 2018) **(C)** Preparation and characterization of cyclodextrin-(CD), EHL, (CD-EHL): (a) Schematic diagram of β-CD conjugated with EHL; (b) molecular weight distribution of EHL and CD-EHL; (c) 1H-NMR spectra of 6-TsO-β-CD, EHL and CD-EHL; (d) infrared spectrograms of EHL and CD-EHL (Zhou et al., 2019). **(A)** Copyright 2012 Wiley **(B)** Copyright 2018 Elsevier **(C)** Copyright 2019 MDPI.

Gatifloxacin (GFLX) is a modern fluoroquinolone with good antibacterial activity and broad antimicrobial properties (Miyai et al., 2008). Water-insoluble adriamycin (DOX) and adriamycin hydrochloride (DOX-HCl) are widely used chemotherapeutic agents for the treatment of cancer (Zhang et al., 2012) (Figure 10A). Chen et al. (2018) selected microwater-soluble GFLX, water-soluble DOX-HCl and water-insoluble DOX to investigate the drug-carrying and drug-release properties of the prepared LNCs (Figure 10B). The results showed that the lignin nanoparticle LNCs (APS-LNCs) prepared with aqueous sodium p-toluenesulfonate (APS) showed good loading capacity to GFLX, DOX-HCl and DOX. The encapsulation efficiency (EE) of APS-LNCs showed a trend of increasing and then decreasing with the initial concentration of lignin. The EE of APS-LNCs was highest at an initial concentration of 50 g/L lignin. In contrast, for the water-soluble DOX-HCl and APS-LNCs, there was a higher EE (42.6%) at a lower initial concentration (1 g/L) due to the strong interaction of the positively charged DOX-HCl with the negatively charged LNCs. These studies broaden the application of high-value lignin and make an irreplaceable contribution in this field.

Zhou et al. (2019) grafted β-cyclodextrin (β-CD) with a hollow ring structure onto EHL to form CD-EHL. Hollow nanoparticles (LHNPs) encapsulating the antitumour drug hydroxycamptothecin (HCPT) were then prepared by self-assembly of modified lignin (Figure 10C). The results showed that β-CD improved the network structure of the modified lignin molecules. In addition, the LHNPs self-assembled using CD-EHL had larger specific surface area and greater porosity, and exhibited hollow structure and stability in phosphate buffered salt solution. The drug loading and encapsulation rates of HCPT were 70.6 ± 9% and 22.02 ± 2%, respectively.

Since the LCST of conventional LCST-type polymers (NIPAM) is 32°C, when the drug is self-assembled and encapsulated by conventional LCST-type polymers, the drug shrinks at 37°C and cannot be released in the human body. To solve this problem, Liu W. et al. (2022) coated DES lignin with aspirin *via* ethanol/water self-assembly to obtain aspirin-lignin (Aspirin@LTNP) nanoparticles. To facilitate the release of aspirin@LTNP *in vivo*, a lignin-based thermosensitive polymer with an LCST of 41.35°C was used as a carrier and released *in vitro* at a rate of 73.75 ± 1.16%. It could be completely explained that when the temperature was lower than 41.35°C, the nanoparticles dissolved in the solution and showed the trend of expansion, the molecules showed the tensile state, and the permeability showed the trend of increase with the increase of temperature. The optimal release rate of aspirin reached 73.35 ± 1.16% at 37°C, and aspirin could be released from the human nanoparticles when the body temperature was not higher than 40°C. In addition to this, the paper went on to explore the effect of pH on drug release. It was found that there was a definite trend at pH 1.5 and pH 7.4 during the first 4 h. However, as time progressed, the cumulative release rate at pH 1.5 was progressively higher than that at pH 7.4 due to the slightly larger particle size of aspirin@LTNP in the strong acid (526.9 nm) than in the weak base (500.3 nm) and the stronger solubility of aspirin in the strong acidic solution than in the weak base solution. The cumulative release rate at pH 1.5 was progressively higher than that at pH 7.4, with the cumulative release rate increasing slowly with time. In the absence of grafted pH-sensitive monomers, the nanoparticles exhibited pH-sensitive response properties at different pH values (1.5 and 7.4).

### 5.2 Cosmetics

**FIGURE 11:**
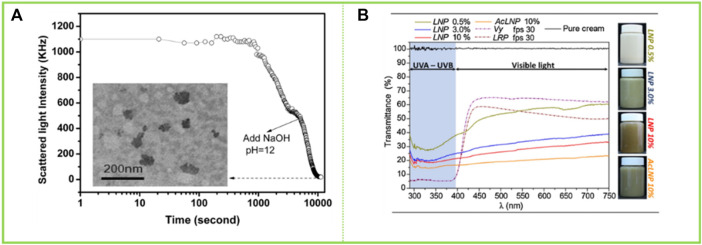
**(A)** Scattered light intensity of the ACL colloidal suspensions with addition of NaOH as a function of storing time (Qian et al., 2014b) **(B)** UV–vis transmittance of neutral creams blended with LNP (0.5, 3.0 and10 wt%) and ACLNP (10 wt%) in comparison to commercial sunscreens with fps 30 (LRP = La Roche-Posay® and Vy = Vichy Laboratories®) (Trevisan and Rezende, 2020). **(A)** Copyright 2014 Royal Society of Chemistry **(B)** Copyright 2020 Elsevier.

Lignin has been of interest as a natural product with free radical scavenging capabilities. Qian et al. (2015b) reported on the potential of using lignin in sunscreens as cytotoxicity tests proved it to be safe. LNCs are an important improvement for the field of skin health given their high specific surface area, which can enhance antioxidant properties, etc. Qian et al. (2014b) prepared LCS of different sizes and structures by self-assembly, which were then mixed with pure skin creams to develop lignin-based sunscreen creams. The creams with added LCS had better sunscreen properties compared to those with added lignin due to the fact that the lignin became more homogeneous and smaller in size (particle size around 50 nm) during the self-assembly process to form the colloids (Figure 11A). Phenolic hydroxyl groups are known to play an important role in the Sun protection process of lignin, and a large number of phenolic hydroxyl groups are exposed during the formation of LCS without any chemical structural changes, which contributes to a significant increase in the Sun protection factor (SPF) of the sunscreen prepared with the addition of LCS, with the SPF of sunscreens containing 10 wt% LCS being increased to 47.71. Trevisan and Rezende (2020) acetylated lignin isolated from elephant grass and self-assembled into LNCs in an acetone solution, which were then mixed with a neutral water-based cream that showed a lower transmission rate in the visible region than commercial sunscreens. In addition, the visual effect of sunscreen emulsions prepared at controlled concentrations in the range of 0.5%–10% was found to be similar to that of tinted creams in the study (Figure 11B). Such applications of lignin have been attracting the attention of experts and scholars, and this paper reviews again to make a contribution to the next.

### 5.3 Emulsification

**FIGURE 12:**
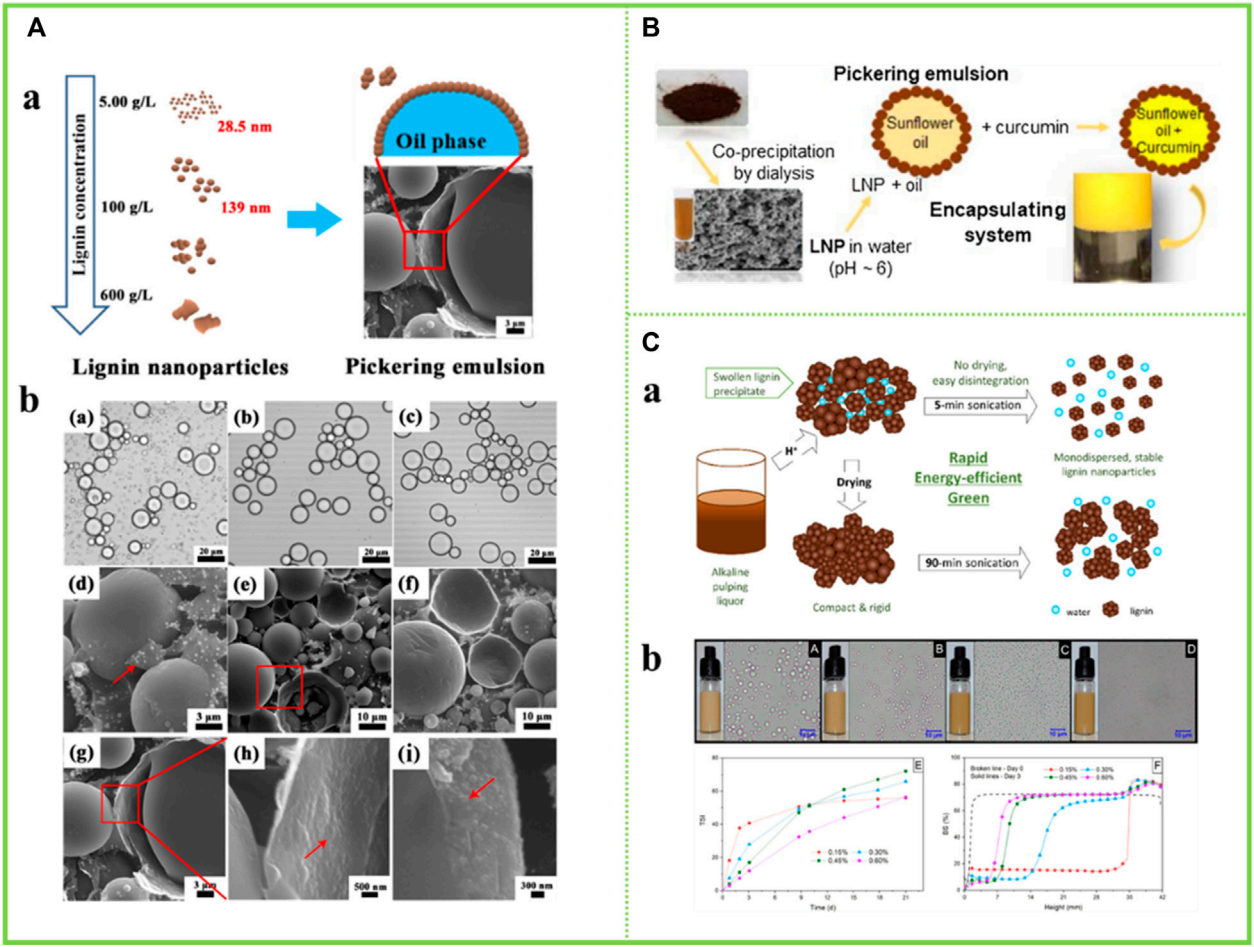
**(A)** Synthesis and application of LNCs, O.M. images of Pickering emulsions of thyme oil in water stabilized (Li et al., 2021) **(B)** An illustration of LNCs-stabilized Pickering emulsions (Qian et al., 2015a) **(C)** Application of particles in Pickering emulsion (Agustin et al., 2019). **(A)** Copyright 2021 American Chemical Society **(B)** Copyright 2015 Elsevier **(C)** Copyright 2019 American Chemical Society.

Recently, LNCs have been considered as a promising alternative to stabilize Pickering emulsions (Bai et al., 2019). Li et al. (2021) used size-tunable LNCs obtained in an acetone and water system to emulsify thyme oil and water systems to form stable oil-in-water Pickering emulsions. The obtained emulsion has strong stability, and the size of the liquid drop is changed along with the usage amount of the LNCs and the oil-water ratio. In this paper, for thyme oil, emulsions can be formed at oil/water ratios as high as 5:5 (v/v) and at lignin nanoparticle additions as low as 0.05 wt% (Figure 12A). This paper is a step forward in the controlled preparation of LNCs for Pickering emulsion applications. Bertolo et al. (2019) used the prepared spherical colloidal LNCs as stabilizers for Pickering emulsions as encapsulants for curcumin (Figure 12B). The LNCs prepared in organic solvents were found to be the most effective stabilizers, retaining 73% of curcumin in their encapsulated form after 96 h. Therefore, this study demonstrates the potential of nanostructured lignin for biomedical applications. Agustin et al. (2019) used LNCs as emulsifiers to stabilize the rapeseed oil and water system to form stable oil-in-water Pickering emulsions and explored the stability of the emulsions over a 21-day period. It was found that the stability of the emulsion increased with the increase of lignin concentration, and the trend was significant in the first week after the preparation of the emulsion. Besides, the stability index (TSI) value of the emulsion with the solid content of LNCs of 0.15 wt% tended to be stable (Figure 12C).

## 6 Conclusion

Lignin is increasingly becoming a hot topic of research as an important biomass resource, and deserves to be valued as a strategic resource at a time when fossil resources are becoming increasingly strained. We have observed an increasing number of studies focusing on the dismantling and utilization of lignin macromolecules, including catalytic degradation, filler materials or composite materials. Despite the increasing trend towards their use, the key scientific issues have not yet been effectively addressed. The main problems include: 1) the sub-molecular structures of lignin macromolecules contained in different plant species; 2) the intramolecular/intermolecular interactions and linkage forms of the sub-molecular structures; 3) the solubility and dispersion of different lignin species in nearly absolute mono-solvents and the self-assembled structures in multi-scale complex dispersion systems; 4) the selective disassembly and screening of lignin macromolecules; 5) the establishment of general synthesis methodologies. In this review we summarize and comment on the above concerns, with particular attention to the macromolecular structural composition of industrially commonly used species of lignin; the assembly morphology of these lignin molecules in mono- or multi-solvents; the preparation of functional substance composite LNCs, and the application of lignin composite nanoparticles in therapy, cosmetics and emulsification applications. Through this systematic review, we gain an insight into the basic theory and engineering applications of lignin and look forward to the futuristic development of lignin.

With the advent of the “biomass era”, the development of lignin will be more focused in terms of depth and breadth: 1) multi-scale modelling of lignin macromolecule composition and assembly of complex systems; 2) conformation and aggregation of lignin macromolecules in near-absolute mono-solvents, *in situ* observation and kinetic topology evolution; 3) targeted lignin macromolecule general methods for selective splitting and extraction of lignin macromolecules; 4) order-constrained regulation of lignin macromolecules during solubilization and assembly; 5) general methods for the preparation of nanoscale lignin hybrid particles, etc. Therefore, lignin has a bright future and is expected to play more excellent roles in all life cycle activities of all living organisms.
